# Improving Taxonomic Delimitation of Fungal Species in the Age of Genomics and Phenomics

**DOI:** 10.3389/fmicb.2022.847067

**Published:** 2022-02-17

**Authors:** Ashley Stengel, Kimberly M. Stanke, Amanda C. Quattrone, Joshua R. Herr

**Affiliations:** ^1^Complex Biosystems Interdisciplinary Life Sciences, University of Nebraska-Lincoln, Lincoln, NE, United States; ^2^Department of Agronomy and Horticulture, University of Nebraska-Lincoln, Lincoln, NE, United States; ^3^Department of Plant Pathology, University of Nebraska-Lincoln, Lincoln, NE, United States; ^4^Department of Chemical and Biomolecular Engineering, University of Nebraska-Lincoln, Lincoln, NE, United States; ^5^School of Biological Sciences, University of Nebraska-Lincoln, Lincoln, NE, United States; ^6^Center for Plant Science Innovation, University of Nebraska-Lincoln, Lincoln, NE, United States

**Keywords:** fungal diversity, eco-evolutionary theory, integrative taxonomy, species delimitation, omics, bioinformatics

## Abstract

Species concepts have long provided a source of debate among biologists. These lively debates have been important for reaching consensus on how to communicate across scientific disciplines and for advancing innovative strategies to study evolution, population biology, ecology, natural history, and disease epidemiology. Species concepts are also important for evaluating variability and diversity among communities, understanding biogeographical distributions, and identifying causal agents of disease across animal and plant hosts. While there have been many attempts to address the concept of species in the fungi, there are several concepts that have made taxonomic delimitation especially challenging. In this review we discuss these major challenges and describe methodological approaches that show promise for resolving ambiguity in fungal taxonomy by improving discrimination of genetic and functional traits. We highlight the relevance of eco-evolutionary theory used in conjunction with integrative taxonomy approaches to improve the understanding of interactions between environment, ecology, and evolution that give rise to distinct species boundaries. Beyond recent advances in genomic and phenomic methods, bioinformatics tools and modeling approaches enable researchers to test hypothesis and expand our knowledge of fungal biodiversity. Looking to the future, the pairing of integrative taxonomy approaches with multi-locus genomic sequencing and phenomic techniques, such as transcriptomics and proteomics, holds great potential to resolve many unknowns in fungal taxonomic classification.

## Graphical Abstract

Enhanced resolution of spices boundaries.

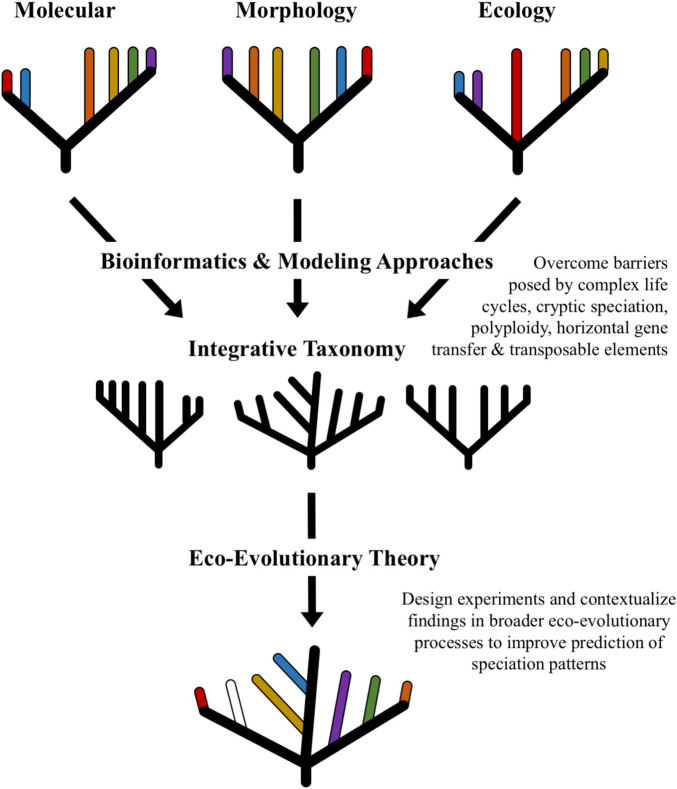

## Introduction: Species Concepts and the Role of Eco-Evolutionary Dynamics

**FIGURE 1:**
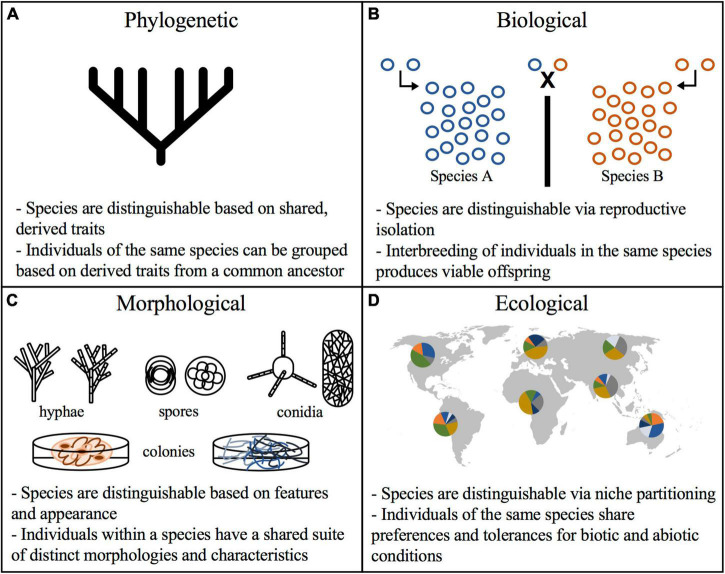
Species concepts criteria for fungal delimitation. Predominant species concepts and the features used to distinguish species. **(A)** Phylogenetic species concept relies on parsimony in reconstructions of taxa relatedness and groups organisms by shared traits; **(B)** biological species concept draws boundaries based on reproductive isolation wherein organisms incapable of producing viable offspring are considered separate species; **(C)** morphological species concept distinguishes species using macro and micro morphology, including colony appearance, pigmentation, conidia, mycelia, and spores; **(D)** ecological species concept evaluates biogeographical ranges and potential niches to delimit taxonomic groups.

The overarching goal of species concepts is to provide a framework for the effective and reliable classification of organisms into logical categories. Decades of discussion have given rise to several predominant species concepts (Figure 1; reviewed in Zachos, 2016). Most notably, these include concepts based on biological (Mayr, 1996), phylogenetic (Nixon and Wheeler, 1990; Taylor et al., 2000; de Queiroz, 2005), morphological (Michener, 1970; Bérubé and Dessureault, 1989; Nakasone, 1996), and ecological criteria (Van Valen, 1976; Schmidt et al., 2014). These concepts have been recently reviewed by Xu (2020), addressed for plant pathogenic fungi by Cai et al. (2011), and applied to Arbuscular mycorrhizal fungi (AMF) by Bruns et al. (2018). Overall, there is growing recognition that a single set of criteria do not sufficiently describe the diversity seen among fungal lineages.

In the last 15 years, efforts have been made to shift the discussion of species delimitation away from a preset list of criteria and toward an understanding of the community-level processes driving patterns of diversification (de Queiroz, 2007). In fact, the General Lineage Concept (de Queiroz, 2005) arose to highlight the role of history (i.e., evolution) and circumstance (i.e., environment) in driving differences between the contingent properties of species. Through this framework, evolutionary groups are distinguishable due to their shared history and processes of adaptation and genetic drift. These eco-evolutionary processes give rise to contingent properties that scientists may use to trace and separate species (de Queiroz, 2007). In recent years, discussion of eco-evolutionary dynamics (Brunner et al., 2019) and its application to microbial communities (Saikkonen et al., 2020) have gained attention. Eco-evolutionary dynamics recognize the role of ecological properties (e.g., spatial heterogeneity, habitat conditions, dispersal, and assembly patterns) and evolutionary trajectories (e.g., life history traits, co-evolution mosaics, intra- and inter-population selection) in reciprocally influencing one another (Brunner et al., 2019). As such, applying an eco-evolutionary framework to microbial interactions has offered new insight into the processes shaping communities and species interactions (McDonald et al., 2020). For example, the framework has given rise to the coupling of molecular and microbiological tools with theoretical approaches toward genetics, phenotypic evolution, species co-evolution, and ecology (Saikkonen et al., 2020). This has improved the identification of keystone taxa, the elucidation of processes shaping host-microbe interactions, and the assignment of putative functions to communities (Cordovez et al., 2019). With regards to fungal species, eco-evolutionary dynamics offer a conceptual framework for researching core ecological and evolutionary processes yielding insight into patterns of biological diversity that shape metapopulations, and ultimately improve the delimitation of species boundaries.

Similar to eco-evolutionary theory, integrative taxonomy is a conceptual framework that recognizes the roles of ecological and evolutionary processes in shaping species boundaries. Therefore, applying recent advances in eco-evolutionary frameworks for microbiome diversity with integrative taxonomy approaches offers great potential for improving research design and interpretation. In practice, integrative taxonomy aims to combine discipline specific observations with broad technical evidence to develop more holistic conclusions regarding evolution and species delimitation (Dayrat, 2005). This approach builds on coalescent theory (Avise and Wollenberg, 1997), combining criteria from various species concepts (e.g., biological, phylogenetic, morphological, etc.) in order to delimit taxa in a way that bridges disciplines and methods of study. Integrative taxonomy has also been referred to as a “multisource approach” (Schlick-Steiner et al., 2010) and has been applied across several eukaryotic lineages, with recent examples including plants (Prata et al., 2018), insects (Lamarre et al., 2016), and fish (Pugedo et al., 2016). Using eco-evolutionary theory to contextualize integrative taxonomy approaches will enable hypothesis testing which can improve discrimination of genetic, phenotypic, and functional traits that span the fungal tree of life. To better understand the utility of integrative taxonomy and eco-evolutionary framing for research, we highlight how key features of fungal biology have posed challenges for taxonomic delimitation and study. Keeping these challenges in mind, we then address how advances in sequencing, “omics,” bioinformatics, and modeling can be applied within the eco-evolutionary and integrative taxonomy frameworks to resolve fungal species boundaries.

## Challenges to Defining Fungal Species

### Complex Life Cycles and Cryptic Species

One of the major obstacles facing the delimitation of fungal species is variation in morphology. The complexity and diversity in life cycles exhibited by fungi has led researchers to misidentify species in two major ways–through dual-naming conventions or cryptic species (Taylor, 2011). The type species described for the sexual stage (teleomorph) and the asexual stage (anamorph), may be very morphologically different. Molecular analyses have shown seemingly disparate taxa to represent the same species. One such example is that of *Curvularia*–an important species complex of phytopathogens that are destructive largely in grasses and cereal plants. This group of filamentous fungi have a *Cochliobolus* teleomorphic stage that has resulted in several changes in taxonomy as well as confusion with a related fungal genus *Bipolaris* (Manamgoda et al., 2012). Similarly, medically relevant fungal organisms may exhibit different biochemical and morphological traits in their anamorph-teleomorph phases, which has led to difficulty in identifying clinically relevant strains (Latouche et al., 1997). Detecting species across life stages in complex cycles is crucial for capturing biodiversity among fungal lineages and improving our understanding of eco-evolutionary pressures that drive natural variation among populations.

A second obstacle is that fungal species may be easily misidentified when two specimens exhibit identical morphologies and/or very closely related phenotypic traits yet show clearly distinct genetic profiles. Often termed “cryptic species,” this moniker highlights that species boundaries in fungi are not always clearly defined (Bickford et al., 2007). *Fusarium subglutinans*, which has more than 20 individuals described as part of the species complex, is a group of well documented plant pathogens demonstrating similar morphology, but distinct biological, ecological, and phylogenetic relationships (Dugan and Everhart, 2016). Similarly, recent evidence shows that *Fusarium* species causing Fusarium Head Blight disease in wheat and other grains are capable of shifting geographic ranges or altering mycotoxin production, which poses a significant challenge to crop yields worldwide (Valverde-Bogantes et al., 2020). Another important example includes the *Aspergillus niger* segregates, which display differing metabolite production, diverse preferential environmental niche adaptation, and broad host ranges despite highly similar and at times indistinguishable morphological characteristics (Howard et al., 2011). Improving the resolution of species boundaries is important for elucidating environmental preferences and evaluating the potential for toxin production which are essential for developing effective disease management practices.

### Polyploidy and Transposable Elements

Polyploidy arising from partial and whole genome duplication events (e.g., auto-polyploidy) has been widely studied in fungi. While specific mechanisms and functional relationships are not fully characterized, there is evidence suggesting that fungi may gain some benefits from harboring larger genomes. For example, changes in ploidy can increase organismal fitness during periods of stress and may facilitate acclimatization to changing environments (Todd et al., 2017). *Rhizophagus irregularis* demonstrates higher densities of poly allelic single-nucleotide polymorphisms, contributing to high within-isolate variability (Wyss et al., 2016). This could be attributed to heterokaryotic states among the population that give rise to differing gene copy numbers and divergence in copies among isolates (Tisserant et al., 2013). This variation further complicates that ability to draw distinctions between isolates and clearly demarcate species boundaries.

Polyploidy can also arise through the merging of genomic content between different species, called allopolyploidy. An evaluation of more than 600 genome assemblies from fungal proteomes and genomes revealed that transposable elements cluster together and contribute to larger fungal genomes (Muszewska et al., 2019). In plant pathogenic fungi, horizontal gene transfer and horizontal chromosome transfer has been observed as a means to expand host ranges (Mehrabi et al., 2011). Furthermore, aneuploidy events can cause the loss of transposable elements which in turn influence fungal lifestyle. The strong selection pressures for and widespread presence of ploidy among fungal lineages pose significant challenges to identifying and characterizing species. Furthermore, the mechanisms driving changes in ploidy are not completely understood. This gap in knowledge draws attention to the importance of developing robust methods that can delimit species in a reliable way.

## Emerging Techniques and Strategies

### Multi-Locus Sequencing

In addition to their contribution to genomics, the advent of high-throughput sequencing approaches has brought about many advances in the way we study fungal communities, particularly those found in natural and agricultural environments. Recent reviews have discussed the role of DNA sequencing to identify bacteria and fungi in natural communities (Inderbitzin et al., 2020), as well as sampling and laboratory protocols, and analysis techniques to garner more reliable insight into mycobiome diversity (Nilsson et al., 2019). Tekpinar and Kalmer (2019) review various molecular markers and assess their utility in fungal identification and phylogeny construction, concluding that the ITS operon can capture a high degree of interspecies variability. However, sequence variation within the same genome can pose a significant challenge for correctly assigning phylogeny to ITS rRNA sequences, as is the case for many arbuscular mycorrhizal fungi (AMF), including *Glomus intraradices* (Börstler et al., 2008). For AMF phylogenies the ITS operon has underperformed in comparison to 18S rRNA (Hart et al., 2015) due to high sequence length variability (Reich and Labes, 2017) and low sequencing depth (Berruti et al., 2017). As such, the use of the ITS operon in combination with protein-coding genes may be more effective for species-level identification of fungi (Lücking et al., 2020), including AMF (Wyss et al., 2016). The multi-gene or multi-locus sequencing approach has gained attention in distinguishing fungal taxa at a finer scale, thereby allowing hypothesis testing in an evolutionary context that also acknowledges phenotypic characters (Matute and Sepúlveda, 2019). This concordance approach, also called phylogenomics, allows different phylogenetic trees to be constructed based on the combinations of genes used to infer phylogenetic relationships (Herr et al., 2015; Hibbett et al., 2016).

**FIGURE 2:**
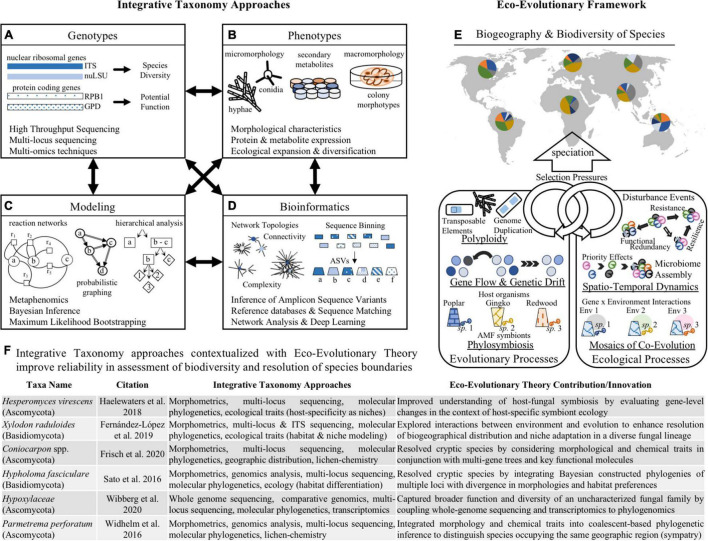
Integrating conceptual frameworks and methodologies for improved fungal species delimitation. The combination of employing integrative taxonomy approaches **(A–D)** with the conceptual framing of eco-evolutionary theory **(E)** can lend new insight into **(F)** prior research and provide a potential scaffold for advancing the study of fungal species. The left side shows various methodological strategies that enable application of the integrative taxonomy framework to resolve species boundaries among fungi. These strategies highlight the relevance of evaluating functional traits of fungal organisms, such as **(A)** genotype data including high-throughput sequencing of one or more gene regions associated with nuclear DNA or protein-coding regions; and **(B)** phenotypic information that describes life strategies including morphological characteristics, protein and metabolite expression profiles, and biogeographical distributions for inferring species ranges and boundaries. Further, applying emerging computational and mathematical techniques to functional trait analysis can enhance resolution of species boundaries, such as **(C)** bioinformatics approaches with improved binning of Amplicon Sequence Variants (ASVs) to more accurately assign taxonomy, and network analysis with improved discrimination of community-level interactions; and **(D)** modeling approaches that employ statistical foundations to distinguish species using graph theory, reaction networks, and Bayesian inference using priors. The right side highlights the role of eco-evolutionary theory in enhancing resolution of fungal species boundaries. This conceptual approach allows for improved design of research studies and interpretation of experimental results by considering **(E)** the reciprocal and interacting roles of ecological and evolutionary processes on shaping the diversity and distribution of species. Looking at **(F)** specific examples of prior research using integrative taxonomy approaches to resolve fungal species boundaries, we can apply eco-evolutionary theory to these findings to provide a scaffold for carrying out and describing future research.

A major advantage to using multi-locus phylogenetic analysis is the ability to resolve cryptic species. This is largely due to the higher degree of homoplasy among closely related cryptic species that enables haplotype-level discrimination not feasible between more distantly related taxa (Sato et al., 2020). Among closely related groups, the overall variation across the ITS regions may either not be enough or be too much for accurate discrimination, therefore, incorporating additional genetic information may improve partitioning between lineages. Examples of improved delimitation using multi-locus sequencing extend across fungal phyla and fungal lifestyles (Somma et al., 2019; Li et al., 2020; Nie et al., 2020). In fact, parallel sequencing of single copy nuclear genes and mitochondrial (mtLSU, mtSSU) and nuclear (ITS1, ITS2) ribosomal regions was able to distinguish haplotypes of the wood-decomposing Basidiomycete *Hypholoma fasciculare* species complex (Sato et al., 2020). Similarly, concordance analysis based on whole genome sequencing indicated the existence of three distinct host-specialized species of ant-pathogenic fungi from the cryptic species complex *Ophiocordyceps unilateralis* (Kobmoo et al., 2019). Expanding the pool of genes used for phylogenetic reconstruction is an important strategy for improving our understanding of evolutionary relationships between fungal species, including resolving unknown signals within cryptic species complexes (Figure 2A).

### Omics and Modeling

The emergence of species along evolutionary trajectories is shaped by a suite of stochastic processes (e.g., random mutations, chance events, population size, modes of reproduction, selection pressures from abiotic factors, etc.) that may give rise to distinct genetic or phenotypic signatures (Figure 2E). As such, meta-“omics” techniques (i.e., genomics, transcriptomics, proteomics, metabolomics) provide another avenue for discovering the breadth of fungal biodiversity and for drawing clearer species boundaries (Kuske et al., 2015). These strategies are especially useful for overcoming the challenges posed by polyploidy and cryptic species, as elucidation of the genetic and functional traits of organisms provides greater insight into phylogenomic and eco-evolutionary patterns (Martiny et al., 2015).

Metagenomic and whole-genome sequencing approaches enable researchers to evaluate phylogenetic relationships using comparative and consensus-based strategies (recently review by Xu, 2020). This has clarified mis-assignment of fungal species with dual naming conventions based on morphological differences between anamorph/teleomorph stages (Taylor, 2011), and improved discrimination of cryptic species by providing robust molecular data that accounts for stochastic processes like gene flow (Kobmoo et al., 2019). Additionally, whole-genome datasets may be evaluated with population genomic approaches to build coalescent phylogenies from a suite of nuclear loci, which improves the accuracy of cryptic species delimitation (Sato et al., 2020). Similarly, metatranscriptomic data captures a large subset of genes from the active members of microbial communities that may be used to build coalescent phylogenies capable of detecting strain-level differences. Marcelino et al. (2019) showed how metatranscriptomics was able to correctly classify fungal taxa living in mixed communities and further identify strain-level variation within the cryptic species complex *Cryptococcus*. The evaluation of the secretome–or the secreted proteins differentiated from proteomic data–is an emerging technique that enables the detection of pathogenic fungi associated with human and animal hosts (Varona et al., 2020). The expansion of multi-“omics” techniques–using a combination of genomic and phenomic data–has further enabled the application of phylogenomic coalescent approaches to improve delineation of fungal species living in diverse environments (Reich and Labes, 2017; Libkind et al., 2020; Wibberg et al., 2020). Despite barriers posed by cryptic speciation, horizontal gene transfer between species, and genome plasticity, contemporary genomic and phenomic methods are able to resolve ambiguity in species boundaries. In particular, coupling genome-based techniques with exploration of the phenome, including expression profiles (e.g., transcripts, proteins, metabolites), morphological characters, and biogeographical ranges, offers new insight into the ecological and evolutionary processes that drive speciation in fungi (Martiny et al., 2015; Reich and Labes, 2017). These techniques highlight how integrative taxonomy approaches may provide deeper insight into drivers of evolution that shape species boundaries (Figures 2A,B). These findings can be better conceptualized using the eco-evolutionary framework to address how evolutionary trajectories are modified by and in turn influence fungal ecology.

Beyond emerging laboratory and measurement techniques, advancements in modeling and applied mathematics also offer additional insight into identification of speciation in fungi (Figure 2C). For example, Bayesian inference and maximum likelihood phylogenetic reconstruction are useful for building coalescent trees to resolve species boundaries, particularly when used in conjunction with multi-locus molecular data (Widhelm et al., 2016; Wyss et al., 2016). Further, metaphenomic modeling (Chowdhury et al., 2019) evaluates microbial phenomics using a reaction network graphical modeling approach to predict the impact of environmental variables on microbial community transcriptional and metabolic expression. Identifying how patterns of microbial signaling and expression change along environmental and ecological conditions provides important insight into potential drivers of microbial diversity and evolution. An additional emerging modeling technique uses human-like decision making, rather than standard 0 or 1 (i.e., “true” or “false”) binary systems. This paradigm, termed fuzzy logic, has been used by Yusof et al. (2013) in an automated wood species recognition system to pre-classify tropical wood species in timber industries, improving accuracy by over 4%. Overall, this may better capture evolutionary trajectories which do not follow binary, deterministic processes. These and other modeling techniques may offer important insight into delineation of fungal species, particularly when used in combination with eco-evolutionary theory to contextualize modeling outputs.

### Bioinformatics and Computational Biology Approaches

The emergence of novel data-analysis and bioinformatic approaches have made statistical investigation into phylogenomics and population genetics more feasible (Figure 2D). For example, random forest classification of fungal ITS barcodes using the ITS2VEC software program makes species identification easier for large datasets as it relies on dimensionality reduction algorithms (Wang et al., 2020). Similarly, in the study of human-associated fungal communities, the software program HumanMycobiomeScan (Soverini et al., 2019) enables direct analysis of metagenomic reads without any pre-processing steps to detect and extract fungal sequences. Improved access to computational techniques allows life science researchers to apply complex mathematics and bioinformatic approaches without extensive knowledge of computational coding.

Further, deep learning and network-based approaches are being employed to classify and cluster fungal sequences from metagenomic data (Vu et al., 2020). These approaches, developed with the implementation of convolutional neural networks, quickly and accurately assign taxonomic ranks, outperforming more traditional nucleotide read identification methods such as BLAST (Altschul et al., 1990) and RDP (Wang et al., 2007). Improved clustering methods contributes to more reliable characterization of fungal biodiversity across diverse systems. A recent analysis of bioinformatics software showed that the choice of data analysis pipeline influences the accuracy and reliability of taxonomy assignment in plant and soil associated fungal communities (Pauvert et al., 2019). The authors of that study conclude that detection of Amplicon Sequence Variants (ASVs) using DADA2 software (Callahan et al., 2016) outperforms more than 350 other software parameter combinations, yielding higher species richness and more detailed community composition data for fungi (Pauvert et al., 2019). New bioinformatics tools are providing novel and innovative strategies for classifying organisms from complex communities, thereby improving understanding of fungal biodiversity and species variability. Improved insight into biodiversity facilitates the development of experiments to evaluate the functional consequences, ecological drivers, and evolutionary trajectories of fungal species from a diverse range natural and agricultural of systems.

## Discussion: Ways Forward

Distinguishing fungal species boundaries is an important practical concern for researchers seeking to address questions related to biodiversity, species interactions, biogeography, ecological processes, and evolutionary dynamics. Conceptually, eco-evolutionary theory recognizes the interplay between ecological processes and evolutionary dynamics in shaping genetic, phenotypic, and functional traits (Brunner et al., 2019; McDonald et al., 2020). Similarly, integrative taxonomy combines technical evidence from many types of observations (e.g., ecological, phylogenetic, morphological, etc.) in order to build coalescent or holistic conclusions (Fujita et al., 2012; Haelewaters et al., 2018; Fernández-López et al., 2019). Together, these two approaches may improve delimitation of species by acknowledging the influence of eco-evolutionary relationships in shaping spatio-temporal variability among and between species (Figure 2). In practice, this may involve using a combination of molecular and morphological techniques to elucidate phylogenetic relationships among cryptic species (Zhou et al., 2014), or to discern causal agents of plant (Kusai et al., 2016) and animal (Haelewaters et al., 2018) diseases. Furthermore, combining multi-locus sequencing with morphological (e.g., spore or conidia measurements) and functional traits (proteome, biochemical compounds, etc.) is improving resolution of diverse fungal groups (Widhelm et al., 2016; Fernández-López et al., 2019; Frisch et al., 2020). Bioinformatics tools, such as amplicon sequence inference (Callahan et al., 2016), and modeling approaches, such as Bayesian hierarchical analysis (Wyss et al., 2016), maximum likelihood probabilistic graphing (Widhelm et al., 2016), and fuzzy logic (Yusof et al., 2013), provide a new toolkit for life scientists to explore the concept of species in fungi. The application of integrative taxonomy strategies to evaluate multiple genotypic and phenotypic factors supports drawing more reliable, robust, and reproducible distinctions between fungal species (Widhelm et al., 2016; Haelewaters et al., 2018; Fernández-López et al., 2019). These approaches are more closely aligned with eco-evolutionary theory, wherein abiotic, biotic, ecological, and environmental interactions contribute to evolutionary trajectories that shape fungal lineages.

Moving forward, we suggest that integrative taxonomy approaches and eco-evolutionary theory will improve research and interpretation of fungal evolution across diverse systems (Figure 2E). Specifically, we propose the use of integrative taxonomy approaches to improve surveys of fungal biodiversity across natural and agricultural systems (Bickford et al., 2007; Möller and Stukenbrock, 2017). This may include exploring spatio-temporal variability among fungal communities in response to environmental stressors (Hawkes and Kiett, 2015; Brooks et al., 2016; Nilsson et al., 2019) and changing climate (Rudgers et al., 2020); as well as investigation of strain-specific responses to perturbations under controlled conditions (Marcelino et al., 2019; Reiter et al., 2020; Varona et al., 2020). These surveys will be particularly important for furthering the understanding of the continuum of symbiosis with applications for human and agricultural disease management (Han et al., 2001; Cai et al., 2011; Kusai et al., 2016). Secondly, we encourage the application of eco-evolutionary theory to conceptualize experimental manipulation of fungal species interactions. Experimental evolution studies are particularly well-suited to explore how fungal communities may shape host health (Koskella and Bergelson, 2020; Morella et al., 2020) and contribute to microbe-microbe interactions that drive speciation (McDonald et al., 2020). Additionally, designing and interpreting research outcomes with concepts such as phylosymbiosis (Kohl, 2020; McDonald et al., 2020) and the geographic mosaic of co-evolution (Medeiros et al., 2018; Fernandes et al., 2019) can improve understanding of drivers of microbial ecology and evolution. This approach is particularly useful for evaluating core microbiomes associated with specific host organisms (Saikkonen et al., 2016; Lofgren et al., 2018), and improving understanding of the functional consequences arising from microbial community interactions (Wei et al., 2019; Trivedi et al., 2020). While our discussion is focused specifically on fungi, many of the analysis techniques and tools are applicable to other microbial lineages, including bacteria and archaea. Thus, it is our hope that the discussion provided here may garner further interest among microbiologists that seek to elucidate the ecological and evolutionary origins of single organisms as well as eco-evolutionary processes shaping entire communities.

## Author Contributions

AS and JH were involved in the initial conceptualization of this manuscript. AS led the literature review and writing of the first draft. AS, KS, and AQ were involved in the visualization of concepts. AS, KS, AQ, and JH provided revisions and additional conceptual input into the manuscript. All authors contributed to the article and approved the submitted version.

## Conflict of Interest

The authors declare that the research was conducted in the absence of any commercial or financial relationships that could be construed as a potential conflict of interest.

## Publisher’s Note

All claims expressed in this article are solely those of the authors and do not necessarily represent those of their affiliated organizations, or those of the publisher, the editors and the reviewers. Any product that may be evaluated in this article, or claim that may be made by its manufacturer, is not guaranteed or endorsed by the publisher.
